# The influence of demographic characteristics, living conditions, and trauma exposure on the overall health of a conflict-affected population in Southern Sudan

**DOI:** 10.1186/1471-2458-10-518

**Published:** 2010-08-27

**Authors:** Bayard Roberts, Eliaba Yona Damundu, Olivia Lomoro, Egbert Sondorp

**Affiliations:** 1 Faculty of Public Health and Policy, London School of Hygiene and Tropical Medicine, 15-17 Tavistock Place, London, WC1 H 9SH, UK; 2 Social and Demographic Statistics Department, Southern Sudan Commission for Census, Statistics and Evaluation, Juba, Southern Sudan; 3 Directorate of Research, Planning and Health Systems Development, Ministry of Health, Government of Southern Sudan, Juba, Southern Sudan

## Abstract

**Background:**

There remains limited evidence on how armed conflict affects overall physical and mental well-being rather than specific physical or mental health conditions. The aim of this study was to investigate the influence of demographic characteristics, living conditions, and violent and traumatic events on general physical and mental health in Southern Sudan which is emerging from 20 years of armed conflict.

**Methods:**

A cross-sectional survey of 1228 adults was conducted in November 2007 in the town of Juba, the capital of Southern Sudan. Multivariate linear regression analysis was used to investigate the associations and relative influence of variables in three models of demographic characteristics, living conditions, and trauma exposure, on general physical and mental health status. These models were run separately and also as a combined model. Data quality and the internal consistency of the health status instrument (SF-8) were assessed.

**Results:**

The variables in the multivariate analysis (combined model) with negative coefficients of association with general physical health and mental health (i.e. worse health), respectively, were being female (coef. -2.47; -2.63), higher age (coef.-0.16; -0.17), absence of soap in the household (physical health coef. -2.24), and experiencing within the past 12 months a lack of food and/or water (coef. -1.46; -2.27) and lack of medical care (coef.-3.51; -3.17). A number of trauma variables and cumulative exposure to trauma showed an association with physical and mental health (see main text for data). There was limited variance in results when each of the three models were run separately and when they were combined, suggesting the pervasive influence of these variables. The SF-8 showed good data quality and internal consistency.

**Conclusions:**

This study provides evidence on the pervasive influence of demographic characteristics, living conditions, and violent and traumatic events on the general physical and mental health of a conflict-affected population in Southern Sudan, and highlights the importance of addressing all these influences on overall health.

## Background

Armed conflict and forced displacement are recognised as important determinants of health [1]. The influence of demographic factors, living conditions, and exposure to violent and traumatic events on specific physical health conditions and mental health conditions such as post-traumatic stress disorder, anxiety, and depression has been well documented [2-7]. However, there is less evidence on the influence of these determinants when a broader conception of health is used which encompasses overall physical, emotional and social well-being rather than specific diseases and conditions. This broader conception of health has the advantage of capturing multiple dimensions of health, rather than perceiving it as merely the absence of disease [8]. The findings from studies applying this broader definition of health can therefore complement those from studies using more narrow and specific outcomes. Only a small number of studies have explored factors associated with the overall health status of conflict-affected populations in low-income countries [9-11], despite this being where the vast majority of conflict-affected persons live [12-14].

This study sought to explore factors affecting overall health in Southern Sudan which experienced a 20 year conflict between the Government of Sudan in the north and rebel movements in southern Sudan led by the Sudan People's Liberation Army/Movement. The conflict was rooted in political, economic and cultural grievances between the south and the Government of Sudan in the north. Approximately 1.9 million deaths from violence, disease and starvation occurred due to the conflict; and up to 5 million people were forcibly displaced from their homes. The signing of the Comprehensive Peace Agreement in January 2005 marked the end of the conflict, with Southern Sudan gaining autonomous status within the Republic of Sudan and a Government of Southern Sudan established. The majority of forcibly displaced persons have now returned to Southern Sudan. However, severe challenges remain in maintaining the peace, ensuring security, fostering political stability and developing economic growth [15-17]. The ability of the Government of Southern Sudan to meet the basic needs and protection of the population is limited, and Southern Sudan characterised by extremely high health needs and very limited availability of health services [18-20]. Two studies have investigated post-traumatic stress disorder and depression and their associated factors in populations in Southern Sudan [21-23]. No published studies could be identified on factors influencing overall health in post-war Southern Sudan. The aim of this study was to investigate the influence of demographic characteristics, living conditions, and violent and traumatic events on overall physical and mental health in Southern Sudan.

## Methods

### Location, study design and sampling method

The study took place in November 2007 in Juba Town, the regional capital of Southern Sudan. Juba Town has increased rapidly in size and population since it was designated as the capital of Southern Sudan after the 2005 Comprehensive Peace Agreement. A conservative population estimate of Juba at the time of this study was 150,000 people and this was rapidly increasing with people moving to Juba in the search for more economic opportunities, security and better access to education and health services. This includes many returning refugees, and there are also a high number of internally displaced persons (IDPs) who fled to Juba during the war and also those fleeing from the continuing insecurity elsewhere in Southern Sudan after the end of the war. As a result of this migration and limited resources and infrastructure, there are a number of unofficial makeshift settlements in Juba - particularly for IDPs which are characterised by inadequate shelter, overcrowded conditions, and poor access to clean water and adequate sanitation.

A cross-sectional survey design was used. The sampling population included adults aged 18 years and over living in Juba Town. The sample size required adequate power (80%) to detect conceptually important differences (0.8 standard deviation) in the health outcomes of different respondent groups within a multivariate analysis with a significance level of 5% with the size of the smallest sub-group of respondents at 5% [24]. Due to the cluster sampling method used, a design effect of 2.0 was included which doubled the required sample size [25]. The expected proportion of unusable questionnaires was set at 20%. The resultant sample size required was calculated to be a minimum of 1160.

A multi-stage cluster sampling method was used as random and systematic sampling methods were not feasible due to limited data on the population of Juba town [25]. The first stage was to randomly select 36 clusters in Juba town. 36 clusters were selected, rather than the more common use of 30 clusters, to reduce the design effect [26]. The 36 clusters were selected using the probability proportional to size method. The sampling frame was a list of the 30 individual bomas (administrative districts) in Juba town with a corresponding running cumulative population size for each boma. This list was from the household count exercise of the census cartography activity of October 2006 which was led by the Southern Sudan Commission for Census, Statistics and Evaluation and considered the most accurate population data available. The clusters were then allocated to bomas proportionally to their population sizes following the probability proportional to size technique to ensure self-weighting [25]. The 36 clusters were allocated to 23 of the 30 bomas in Juba town using this process. The total population living in the 23 bomas was estimated at 131,194 of which 64,416 were estimated to be of eligible age. The sampling process meant that both permanent settlement and more temporary settlements of IDPs, returning refugees and voluntary migrants were included in the survey.

The second stage consisted of randomly choosing individuals from the selected clusters. As the clusters were already self-weighted, the same number of individuals were chosen from within each of the selected clusters. The Expanded Programme on Immunisation (EPI) random walk method was used to help randomly select households for this stage. This involved going to the centre of each boma and then following an imaginary line drawn from the centre to the edge of the boma in a direction determined by spinning a pen. Households along this imaginary line were then counted and a household randomly selected using a random number list. Subsequent households were then selected which were two structures removed from the previously selected structure based on a principal of proximity [26,27].

### Questionnaire

The health outcome of overall health status was measured using the SF-8 instrument developed by QualityMetric. The SF-8 captures multiple aspects of health, it is very brief and so has low burden; and it is relatively easy to translate and adapt to different cultural settings, and has been tested in over 30 different languages and used in a number of countries [28-30], including with IDPs in northern Uganda [31].

The SF-8 uses 8 single-item scales which address 8 domains of general physical and mental health and these items are summarised as follows:

• General Health: How would you rate your health?

• Physical functioning: How did physical health problems limit usual physical activities?

• Role limitation (physical): How much did physical health problems limit your daily work?

• Bodily pain: How much bodily pain have you had?

• Vitality: How much energy did you have?

• Social functioning: How much did physical or emotional problems limit your usual social activities?

• Role limitation (emotional): How much did emotional problems limit your daily activities?

• Mental health: How much have you been bothered by emotional problems?

Each item has a 5 or 6 point response range, and a 4 week recall period was used for the study. It produces continuous summary scores for general physical health and general mental health. These are calculated by weighting each SF-8 scale using norm-based scoring methods and weights derived from the initial validation work for this instrument. Higher summary general physical and mental health scores are indicative of better health [28]. Scores above and below 50 are considered above and below the average in the general U.S. population based upon results recorded by the developers of the SF-8 [28].

The questionnaire also included items on respondents' demographic characteristics (sex, age, marital status, education status); living situation of respondents (availability and sources of drinking water and food, use of household soap, sense of security, access to health services and utilisation of sources of support); and their lifetime exposure to violent and traumatic events including forced displacement using questions adapted from the original Harvard Trauma Questionnaire which has been widely used with conflict-affected populations and also individual questions on forced displacement (frequency, location, current status) [32].

The questionnaire was translated and delivered in Juba Arabic and Bari, the two main languages in Juba town. The translation followed recommended guidelines and was conducted by experienced translators. A review of the forward and back translations and pre-testing was conducted by the study staff to ensure that the meanings and concepts of the questionnaire items remained consistent with the original English version.

### Data collection and data entry

Twenty data collectors were recruited for the survey (11 women and 9 men). They all spoke fluent Juba Arabic and/or Bari and had experience of survey data collection. One week's training was provided for the study. The main data collection took place between 20 and 30 November 2007. All boma leaders and other appropriate administrative officials were informed in advance of the study and provided permission for the data collection. Two data entry clerks were used to enter the data into EpiData Entry version 3.2 (DK-5230, Odense, Denmark). Questionnaires and the dataset were cross-checked by study staff and analysis also conducted of the dataset to check for inconsistent data entries.

### Analysis

The analysis sought to explore factors associated with general physical and mental health using regression analysis (following a stepwise approach with forward selection and backward regression [33]). A univariate linear regression analysis was initially conducted to explore associations of the individual independent variables on the general physical and mental health scores. The individual variables which showed statistically significant (P < 0.05) associations in the univariate analysis were then categorised in to the following three models: (i) demographic characteristics; (ii) living conditions, and (iii) exposure to traumatic violent events. Multivariate analyses were then conducted separately for each of the 3 models to adjust for the influence of the other variables within the same model. The statistically significant (P < 0.05) variables from these separate analyses were then also included in a combined model and multivariate analysis used to adjust for the influence of all the variables included in this combined model. The analysis of the 3 models and the combined model was used to explore the relative influence of the three categories of variables and also their overall influence when combined. Negative coefficients indicate a negative association with the general physical and mental health scores (i.e. worse physical or mental health).

Data quality and the internal consistency of the SF-8 were also assessed. Data quality was firstly assessed by analysing the number of missing responses to individual SF-8 items. A large number of incomplete responses may suggest respondents found the question confusing, inappropriate or uncomfortable to answer [34]. Data quality was secondly assessed by analysing the distribution of item responses of the SF-8 using aggregate endorsement frequencies. For instruments with around a 5 point response range such as the SF-8, any item with 2 or more adjacent response points showing less than 10% of the responses on aggregate are problematic [35]. Internal consistency was analysed to explore the consistency of the SF-8 items with the underlying constructs of physical and mental health by using the Pearson Correlation Test [36-38]. *A priori *hypotheses about the directionality and magnitude of the correlations were made assuming that items more closely related to a common dimension would show a correlation of ≥0.50 [24,39,40]. It was hypothesised that there would exist strong correlations between the general physical health score and items 1-5 (general health, physical functioning, physical role limitation, bodily pain, vitality), and strong correlations between the general mental health score and items 6-8 (social functioning, mental health, emotional role limitation). This test for internal consistency was preferred to other tests for internal consistency such as Cronbach's alpha because the SF-8 contains only a small number of items and these fairly heterogeneous items which cover two different health domains (general physical and mental health).

Statistical significance was assumed for *P *values <0.05. All statistical analysis was performed using STATA version 9.2 (Stata Corporation, College Park, Texas, USA) and adjusted for the clustered design.

### Ethical issues

Ethical approval for the study was provided by the Ministry of Health of the Government of Southern Sudan, and the London School of Hygiene and Tropical Medicine. An information sheet was read out and provided to potential respondents, and a consent form completed to ensure informed consent. All data collected was confidential and anonymous, and securely stored by the study team.

## Results

All 36 clusters in the 23 bomas were visited. There were 32 absent individuals, 13 individuals who did not consent to participate in the study, and 18 questionnaires with incomplete SF-8 items; resulting in 1228 completed interviews (95.1% response rate). A summary of the key characteristics of the study sample are provided in Table 1. Women and men were equally represented in the sample. The mean age of respondents was 33 years. 153 (12.5%) of the respondents were still displaced from their home areas as IDPs. Within the previous 12 months, nearly a third (29.2%) of all respondents had lacked access to food or water and nearly a quarter (24.5%) of respondents had failed to obtain medical care when ill. Over a third (35.9%) of respondents had either previously been forcefully displaced or were still forcefully displaced from their home areas, and 14.6% had ever experienced more than 6 of the 13 violent and traumatic events covered in the questionnaire. The respective mean general physical health and general mental health scores were 42.67 [95% CI 41.84, 43.50] and 44.32 [95%CI 43.59, 45.06], well-below the instrument norm of 50, indicating poor health.

**Table 1:** Sample characteristics (N = 1228)

Characteristics	Number	(%)
**Demographic**		
Number of women	622	(50.7)
**Age**		
Mean age (low-high range)	33 years	(18-81 years)
Age 18-24 years	357	(30.0)
Age 25-49 years	706	(59.3)
Age 50-74 years	121	(10.2)
Age 75+ years	6	(0.5)
*Religion:*		
Christian	1129	(91.9)
Muslim	92	(7.5)
other	7	(0.6)
*Ethnicity:*		
Bari Speaking	605	(49.3)
Muru	148	(12.1)
Madi	72	(5.9)
other	403	(32.8)
*Marital status:*		
married	897	(73.2)
single	248	(20.2)
divorced/separated (includes forcefully separated)	24	(2.0)
widowed	56	(4.6)
*Education level:*		
never attended school	326	26.5
completed or partially primary school	387	31.5
completed or partially completed secondary school	378	30.8
completed post-secondary school (e.g. college)	137	11.2

**Current/recent living conditions**		
no soap currently in household	792	64.7
drinking from unprotected water source	428	34.9
lack food or water (in past 12 months)	358	29.2
lack medical care when ill (in past 12 months)	301	24.5
lack shelter/housing (in past 12 months)	161	13.1
Currently internally displaced persons	153	(12.6)

**Lifetime exposure to violent and traumatic events**		
unnatural death of family/friend	753	59.9
combat situation	632	51.5
murder of family/friend	610	49.7
forcibly displaced (internally displaced or refugee)	441	35.9
close to death	431	35.1
forced separation from family	318	25.9
serious injury	316	25.7
tortured or beaten	248	20.2
forced isolation from others	231	18.8
murder of stranger or strangers	228	18.6
forced to accept thoughts against will	171	13.9
imprisonment	168	13.7
being abducted or kidnapped	102	8.3
rape or sexual abuse	82	6.7

### SF-8 data quality and internal consistency

Eighteen (1.5%) of the completed interviews had 1 or more missing SF-8 item. The response distributions of each item for the sensitivity aggregate endorsement frequency showed all 8 items performing well (Table 2).

**Table 2:** SF-8 scores, item response option distribution, and internal consistency (N = 1228)

SF-8 item		Mean (standard deviation)	Item response option distribution (%)						Internal consistency: item-summary score	
			1	2	3	4	5	6	Mental health score	Physical health score
1	General health	43.27 (9.22)	4.89	14.17	27.28	30.94	12.21	10.50	0.66	0.37
2	Physical functioning	43.43 (10.18)	8.14	14.5	19.22	32.08	26.06		0.79	0.34
3	Role - physical	42.61 (10.41)	7.57	16.12	19.30	27.44	29.56		0.83	0.34
4	Bodily pain	44.88 (11.14)	7.57	18.00	20.03	18.49	18.57	17.35	0.76	0.39
5	Vitality	46.42 (10.27)	4.72	31.35	24.02	26.71	13.19		0.51	0.49
6	Social functioning	44.71 (10.29)	7.17	12.79	23.13	26.63	30.29		0.46	0.65
7	Role - emotional	42.56 (9.38)	5.94	14.01	19.79	28.91	31.35		0.46	0.71
8	Mental health	44.50 (11.12)	8.47	17.35	18.81	28.50	26.87		0.25	0.92

	Summary physical health score	42.67 (10.78)								
	Summary mental health score	44.32 (11.33)								

The results on internal consistency are also presented in Table 2. These show a generally strong level of consistency (≥0.50) of physical health related items (items 1-5) with the general physical health summary score, and mental health related items (items 6-8) with the general mental health summary score. As would be expected, there are weaker correlations of physical health related items (items 1-5) with the general mental health summary score and mental health related items (items 6-8) with general physical health summary score.

### Factors associated with general physical and mental health

The independent variables with statistically significant (P < 0.05) associations with general physical and mental health in the multivariate linear regression analyses are presented in Table 3. The top half of Table 3 ('analyses of separate models') presents the significant variables for each of the three models after adjusting only for the influence of the other variables within the same model. The lower half of Table 3 ('analysis of combined model') presents the significant variables after adjustment for the combined influence of all the other variables from the three models.

**Table 3:** Multivariate analysis of influence of demographic characteristics, living conditions, and violent and traumatic events with general physical and mental health

Model/variable	N	General physical health			General mental health		
		**Coef**.	[95% CI]	*P*	**Coef**.	[95% CI]	*P*
**ANALYSES OF SEPARATE MODELS†**							

**Model 1: demographic variables**							
women	622	-2.41	[-3.49, -1.33]	0.000	-2.61	[-3.74, -1.48]	0.000
increasing age	1228	-0.17	[-0.22, -0.11]	0.000	-0.18	[-0.23, -0.13]	0.000
separated from partner^§^	80	-			-3.10	[-5.33, -0.87]	0.008

**Model 2: living conditions variables**							
no soap currently in household	792	-2.69	[-3.96, -1.41]	0.000	-		
lacked food and/or water*	358	-1.29	[-2.43, -0.15]	0.028	-2.06	[-3.83, -0.29]	0.024
lacked medical care when ill*	301	-3.97	[-5.46, -2.49]	0.000	-3.72	[-5.63, -1.81]	0.000

**Model 3: violent events variables**							
in combat situation	632	-			-1.21	[-2.39, -0.04]	0.044
murder of a family member or friend	610	-			-1.69	[-2.95, -0.44]	0.010
close to death	431	-			-1.92	[-3.40, -0.45]	0.012
serious injury	316	-2.23	[-3.52, -0.93]	0.001	-		
displaced more than once	152	-2.24	[-3.99, -0.49]	0.014	-		
rape or sexual abuse	82	-			-3.36	[-6.61, -0.11]	0.043
more than 6 violent events ‡	179	-3.07	[-5.08, -1.05]	0.004	-3.02	[-4.88, -1.16]	0.002

**ANALYSIS OF COMBINED MODEL††**							

*Demographic variables:*							
Women	622	-2.47	[-3.62, -1.32]	0.000	-2.63	[-3.75, -1.51]	0.000
Increasing age	1228	-0.16	[-0.22, -0.11]	0.000	-0.17	[-0.22, -0.12]	0.000

*Living conditions:*							
no soap currently in household	792	-2.24	[-3.50, -0.99]	0.001	-		
lack food and/or water *	358	-1.46	[-2.59, -0.33]	0.013	-2.27	[-4.11, -0.43]	0.017
lack medical care when ill *	301	-3.51	[-4.85, -2.17]	0.000	-3.17	[-5.06, -1.28]	0.002

*Past violent events:*							
in combat situation	632	-			-1.42	[-2.56, -0.27]	0.017
murder of a family member or friend	610	-			-2.07	[-3.36, -0.77]	0.003
serious injury	316	-1.94	[-3.03, -0.85]	0.001	-		
displaced more than once	152	-1.95	[-3.74, -0.16]	0.034	-		
rape or sexual abuse	82	-			-3.51	[-6.72, -0.30]	0.033
more than 6 violent events‡	179	-2.80	[-4.70, -0.89]	0.005	-2.67	[-4.61, -0.74]	0.008

Abbreviations: CI, confidence interval; Coef., coefficient.

† multivariate analysis conducted separately for variables within each of the models (using statistically significant (*P *< 0.05) variables selected through a univariate analysis).

†† Combined model consists of the statistically significant variables (*P *< 0.05) from multivariate analysis in the three separate models combined into a single model, with multivariate analysis then conducted of all these selected variables together.

§ Separated from partner includes divorced/separated, widowed, forced separation.

- Not included as not statistically significant (at *P *< 0.05) or not relevant.

* Within previous 12 months.

‡ Frequency of trauma events analysed separately from individual trauma exposure variables in multivariate analysis.

In the separate model analyses, the demographics model showed associations of being female and increasing age with lower general physical and mental health scores (i.e. worse health) and being separated from a partner was associated with lower mental health scores. In the living conditions model, lacking food and/or water and lacking medical care when ill were associated with lower general physical and mental health scores; and the absence of soap in the household were associated with lower general physical health scores. In the violent and traumatic events model, serious injury, being displaced more than once, and experiencing more than six violent events were associated with lower physical health scores; and being in combat situation, the murder of a family member or friend, having been close to death, rape or sexual abuse and experiencing more than 6 violent events were all associated with lower mental health scores.

In the combined model analysis, the variable of being separated from a partner was excluded as it was no longer significant. The strength of association of the other demographic variables of being a woman and increasing age with lower general physical and mental health scores remained very similar after adjusting for the effect of the variables in the other two models. In the living conditions model, the variables of absence of soap, lacking food and/or water, and lacking medical care when ill all maintained similar associations with lower general physical and mental health scores. In the violent events model, being close to death was excluded as it was no longer significant. The other trauma exposure variables of being in a combat situation, the murder of a family member or friend, and rape or sexual abuse showed a slightly stronger association with lower general mental health scores. The variables of experiencing serious injury and being displaced more than once showed a slightly weaker association with lower general physical health scores. Experiencing more than 6 violent events had a slightly weaker association with lower physical and mental health in the combined analysis.

## Discussion

This study presents evidence on factors associated with general physical and mental health in a population affected by conflict in Southern Sudan. It also revealed good data quality and internal consistency of the SF-8 with the study population.

The combined multivariate regression analyses resulted in a number of variables covering demographic, living conditions, and violent and traumatic events showing significant associations with general physical and mental health. For the demographic variables, women had significantly lower scores than men in this study. This is also shown in a similar study of IDPs in northern Uganda using the SF-8 [10], and other studies using the SF-8 in stable settings although the differences are not as wide as in this study [28]. A study by Araya *et al *of post-conflict displaced persons in Ethiopia also noted the influence of gender and age on health-related quality of life [9]. The influence of gender on general mental health has been noted in other studies in conflict-affected populations [41-44]. This may be partly attributable to the psychological consequences of rape, the violent loss of partner and children, and of becoming a single parent or widow. Increasing age also showed an association with worse general physical and mental health. The study of IDPs in northern Uganda showed an association of increase age only with physical health. Studies on general mental health have shown an association between increasing age and worse general mental health [43,45,46].

The absence of soap from a household had a negative association with physical health. The association of soap with poor general physical health was also shown in the study with IDPs in northern Uganda [10]. This could possibly be explained by the absence of soap contributing to diarrhoeal disease and worsening physical health [47,48]. Soap may also act as a proxy for other factors not adequately captured in the analysis such as acute poverty or lack of access to clean water and adequate sanitation, and further investigation is required. The lack of food or water in the previous 12 months showed an influence with worse general physical and mental health. The influence of this on mental health could be explained by the uncertainty and anxiety caused by limited income and food insecurity. A study by de Jong *et al *of conflict-affected persons in Kashmir noted that lack of food had an association with worse general mental health [41]. The negative association of illness without access to medical care on general mental health also highlights the strong effect that the absence basic goods and services can have upon mental health, and this was also indicated in the study with IDPs in northern Uganda [10]. The study by Araya *et al *in Ethiopia also noted the effect of poor living conditions on overall health [9]. The findings from this study highlight the need to increase access to essential goods and services such as food, drinking water and health care in Juba.

There is a strong association between both individual trauma events and the frequency of trauma events with poor physical health and mental health. The dose-response relationship between trauma exposure and poor overall health is consistent in the study by Araya *et al *[9], the study of IDPs in northern Uganda [10], and studies on general mental health [41,45,46], as well as specific mental health conditions, including in Southern Sudan [22,23,49]. Importantly, there is increasing concern about the deteriorating security situation in Southern Sudan which could increase exposure to trauma events and further impact upon peoples general physical and mental health [17].

The combined analysis of the variables in all three models showed that the strength of association of the variables with general physical and mental health did not alter substantially, with the exception of two variables (being separated from a partner and being close to death) which no longer showed significant associations. This suggests a strong pervasive influence of the selected demographic characteristics, living conditions, and violent and traumatic events, on general physical and mental health. These findings provide evidence on the importance of addressing all three types of influences on overall health, such as through the provision of services and care for both physical and mental health, the provision of basic goods and services, and adequate security to prevent future exposure to violent and traumatic events.

## Limitations

This study has a number of limitations. First, it did not explore the availability and utilisation of health services and sources of emotional support for general physical and mental health. There is also very little recent data on this in Southern Sudan, particularly for mental health, and further research is required on this. Second, the study did not explore different types of sexual violence such as forced marriage or forced prostitution through separate questionnaire items and so the study results may also not adequately reflect the influence of sexual violence on health. Third, there is a high degree of mobility in the population of Juba town which would have reduced the accuracy of the sampling frame and the sampling process. However, the sampling frame source was felt to be the most accurate data source available. Fourth, further tests for reliability could have been used such as test-retest reliability. In addition, it would have been preferable to have validated the SF-8 against clinical data or validated instruments measuring appropriate health conditions. While this was not possible because of the limited resources available to collect clinical data in the study and the absence of validated health instruments in Southern Sudan, it is recommended that further work should be conducted to validate health measurement instruments in Southern Sudan.

## Conclusions

This study provides evidence on the pervasive influence of demographic characteristics, living conditions, and violent and traumatic events on the general physical and mental health of a conflict-affected population in Southern Sudan, and highlights the importance of addressing all these influences on overall health.

## Abbreviations

CI: Confidence Interval; IDP: Internally Displaced Person

## Competing interests

The authors declare that they have no competing interests.

## Authors' contributions

BR led the study concept and design, data collection, data analysis, and drafting of the manuscript. EYD participated in study development, data collection, and review of the manuscript. OL participated in developing the study development and design, and review of the manuscript. ES participated in developing the study concept and design, and review of the manuscript. All authors read and approved the final manuscript.

## Pre-publication history

The pre-publication history for this paper can be accessed here:

http://www.biomedcentral.com/1471-2458/10/518/prepub
